# Transurethral Incision of the Bladder Neck with or without Additional Procedure Resumes Spontaneous Voiding in Female Voiding Dysfunction—A Long-Term Retrospective Follow-Up

**DOI:** 10.3390/jcm12041514

**Published:** 2023-02-14

**Authors:** Hueih-Ling Ong, Hann-Chorng Kuo

**Affiliations:** 1 Department of Urology, Dalin Tzu Chi Hospital, Buddhist Tzu Chi Medical Foundation, Chia-Yi 622, Taiwan; 2 Department of Urology, Hualien Tzu Chi Hospital, Buddhist Tzu Chi Medical Foundation, Tzu Chi University, Hualien 970, Taiwan

**Keywords:** female, voiding dysfunction, bladder neck obstruction

## Abstract

Aim: This study evaluated the long-term effectiveness of transurethral incision of the bladder neck (TUI-BN) with or without an additional procedure for female voiding dysfunction. Methods: Women with voiding difficulty who underwent TUI-BN in the last 12 years were included. All patients underwent a videourodynamics study (VUDS) at baseline and after TUI-BN. A successful outcome was defined as having a voiding efficiency (VE) increase by ≥50% after treatment. Patients with insufficient improvement were chosen for repeated TUI-BN, urethral onabotulinumtoxinA injection, or transurethral external sphincter incision (TUI-ES). The current voiding status, surgical complications, and additional surgeries were evaluated. Results: A total of 102 women with VUDS evidence of a narrow bladder neck during voiding were enrolled. The long-term success rate of the first TUI-BN was 29.4% (30/102) and increased to 66.7% (34/51) after combining TUI-BN and an additional procedure. The overall long-term success rates were 74.6% in women with detrusor underactivity (DU), 52.0% in detrusor overactivity and low contractility, 50.0% in bladder neck obstruction, 20.0% in hypersensitive bladder, and 75% in stable bladder (*p* = 0.022). Patients with a lower maximum flow rate (Qmax), (*p* = 0.002), lower voided volume (*p* < 0.001), lower corrected Qmax (*p* < 0.001), lower ladder contractility index (*p* = 0.003), lower voiding efficiency (*p* < 0.001), but larger post-void residual volume (*p* < 0.001) had a satisfactory surgical outcome. Spontaneous voiding was achieved in 66 (64.7%) patients, de novo urinary incontinence in 21 (20.6%), and vesicovaginal fistula in 4 (3.9%), all were repaired. Conclusions: TUI-BN alone or in combination with an additional procedure was safe, effective, and durable in patients with DU to resume spontaneous voiding.

## 1. Introduction

Bladder neck obstruction (BNO) in men has been extensively studied, however, BNO in women is often overlooked and under-treated [1]. Previous studies revealed the estimated prevalence of female BNO was between 2.7% and 29% [2,3]. The main reason for the large variation in the prevalence rate is the lack of standard diagnostic criteria for female BNO [4]. Clinically, the complement of lower urinary tract symptoms (LUTS) of in-female BNO is ƒBNO [5].

A videourodynamics study (VUDS) provides a precise diagnosis for female voiding dysfunction [6]. Turner-Warwick et al. described high voiding pressure, low flow rate, and a non-funneling appearance of the bladder neck during the voiding phase in VUDS as bladder neck dysfunction (BND) [7]. However, in clinical practice, the etiology of voiding dysfunction with a narrowing bladder outlet was difficult to diagnose without a VUDS, such as in BND alone or combined with a tight urethral sphincter, or with detrusor underactivity (DU).

Previous studies demonstrated transurethral incision of the bladder neck (TUI-BN) in women with BN obstruction and DU is effective [8,9]. However, the effectiveness in real-world practice has not yet been determined. Moreover, studies on repeated TUI-BN or additional procedures after the first TUI-BN to improve success rates have been scantly reported. This study aimed to evaluate the long-term effectiveness of TUI-BN alone, with repeated TUI-BN procedures, or with an additional procedure in female voiding dysfunction. The predictive factors for a better treatment outcome were also evaluated in this study.

## 2. Materials and Methods

We retrospectively reviewed women who had voiding dysfunction and underwent TUI-BN at least once at our institution from March 2007 to April 2019. All patients underwent VUDS before TUI-BN and after this operation. Female patients with proven urinary tract infections, previous anti-incontinence surgery, or spinal cord injury were not enrolled in this study. This study had been approved by the Institutional Review Board and the Ethics Committee of Hualien Tzu Chi Hospital, Hualien, Taiwan (IRB: 100-06). Informed consent was waived due to the retrospective nature of the analysis.

VUDS was performed in accordance with the recommendations of the International Continence Society [10]. A multichannel urodynamic system (Life-Tech Inc., Stafford, TX, USA) and a C-arm fluoroscope (Toshiba, Tokyo, Japan) were used. VUDS was repeated at least twice to obtain a reproducible pressure flow trace. The first sensation of filling (FSF), full sensation (FS), cystometric bladder capacity (CBC), bladder compliance, maximum flow rate (Qmax), detrusor pressure at Qmax (Pdet.Qmax), post-void residual volume (PVR), sphincter electromyography activity (EMG), voided volume (Vol), bladder outlet obstruction index (BOOI, defined as Pdet.Qmax − [2 × Qmax]), bladder contractility index (BCI, defined as BCI = Pdet Qmax + 5 Qmax), voiding efficiency (VE, defined as voided Vol/bladder capacity × 100%), and corrected maximum flow rate (cQmax, defined as Qmax/Vol^1/2^) were documented. During VUDS, voiding cystourethrography was carried out using a C-arm fluoroscope positioned 45 degrees from the buttocks so that the urethra could be lengthened and the bladder neck, urethral sphincter, and distal urethra could be clearly seen.

Patients with severe difficulty in urination, straining while voiding, large PVR, and a narrow bladder neck observed during voiding in VUDS, were initially treated with alpha-blockers for at least three months. If LUTS were refractory to medication, TUI-BN was advised. The surgical procedure of TUI-BN has been reported in a previous study [8]. If patients still had dysuria, straining while voiding, large PVR, or VE less than 25% after the initial TUI-BN, post-operative VUDS was performed. Consecutive TUI-BN, transurethral external sphincter incision (TUI-ES), or urethral sphincter onabotulinumtoxin A (Botox) injection was advised according to the findings in the second VUDS. Patients with neurological deficit such as a history of cerebrovascular accident, Parkinsonism, myelomeningocele, poliomyelitis, radical hysterectomy, or spine surgery were categorized as with neurologic bladder. Patients without any neurological deficit were grouped into the with non-neurogenic bladder category. The current voiding status was recorded through direct interviews by the same research assistant. The surgical complications such as de novo incontinence and vesicovaginal fistula and additional surgical procedures for complications such as suburethral sling implantation and urethral platelet-rich plasma (PRP) injection were documented.

The descriptions and terminology for urodynamic parameters were in accordance with the recommendations of the International Continence Society [11]. The primary endpoint of this study was the success rate between TUI-BN alone and TUI-BN with an additional procedure. We defined an improvement in VE by ≥ 50% after treatment as a successful outcome. The parameters between the successful and failed groups were compared using the chi-square test for categorical variables and the Wilcoxon rank-sum test for continuous variables. The secondary endpoint of this study is the predictor factors for surgical success. The demographics and VUDS parameters were analyzed using univariate and multivariate logistic regression to clarify the predictive factors and the discriminatory capacity was investigated using an area under the curve (AUC) analysis. A *p*-value < 0.05 was considered statistically significant. Complications of the surgery are the third endpoint for this study.

## 3. Results

The age of patients ranged from 18 to 88 years (mean 61.4 ± 17.4). Among the 102 patients, 64 (62.7%) had a successful outcome and 38 (37.3%) failed the treatment in long-term follow-up. The baseline VUDS identified detrusor underactivity (DU) in 59 patients (57.8%), detrusor overactivity and low contractility (DOLC) in 23 (22.5%), hypersensitive bladder (HSB) in 9 (8.8%), and bladder neck obstruction (BNO) in 4 (3.9%). Among the patients, 44/59 (74.6%) with DU, 13/25 (52.0%) with DOLC, 2/4 (50.0%) with BNO, 2/10 (20.0%) with HSB, and 3/4 (75.0%) with stable bladder were successful (*p* = 0.022). The success rate of DU patients after the first TUI-BN was 30.5% (18 out of 59), and the success rate increased to 76.5% (26 out of 34) in patients who received TUI-BN and an additional procedure. Patients with neurogenic bladder had a higher success rate (73.1%, 38 out of 52) in comparison with non-neurogenic bladder (52%, 26 out of 50) (*p* = 0.028).

The baseline VUDS parameters in the successful group were significantly lower in Qmax, Vol, cQmax, BCI, and VE, but higher in PVR (*p* < 0.05). (Table 1) Significant improvements in the VUDS parameters were noted after surgery in Qmax (5.16 ± 8.53 mL/s), Vol (93.9 ± 169.3 mL), PVR (171.2 ± 239.4 mL), cQmax (0.32 ± 0.55), BCI (17.7 ± 49.3), and VE (0.33 ± 0.42) (*p* < 0.001). (Table 2) Among the 102 patients, 51 underwent TUI-BN alone, 51 patients received TUI-BN followed by an additional procedure, including repeated TUI-BN in 14; TUI-BN and urethral Botox injection in 13; urethral Botox injection alone in 18; TUI-BN and urethral Botox injection and TUI-ES in 3; urethral Botox injection and TUI-ES in 2, and TUI-ES alone in 1.

The entire de novo incontinence was grade 1 stress urinary incontinence and the prevalence rate was 20.6% (*n* = 21), and vesicovaginal fistula 3.9% (*n* = 4). The prevalence rate of de novo stress urinary incontinence was 36.7% (11 out of 30) in patients with TUI-BN alone and 13.9% (10 out of 72) in patients who received TUI-BN and an additional procedure. Among the 21 patients who had postoperative urinary incontinence needing more than 1 pad/day, 12 underwent suburethral sling surgery and 2 underwent urethral sphincter PRP injection. Patients with vesicovaginal fistula received immediate transvaginal fistula repair during the same operation, and all were successfully treated.

The overall success rate was 62.7%, including 29.4% (30 out of 102) with the first TUI-BN alone, and an additional 66.7% (34 out of 51) who had combined TUI-BN with an additional procedure (*p* < 0.001). Among 102 patients, the current voiding status was spontaneous voiding in 66 (64.7%) patients, self-voiding with occasional clean intermittent catheterization (CIC) in 21 (20.6%), CIC dependence in 12 (11.8%), and indwelling a catheter in 3 (2.9%).

A logistic regression univariate analysis showed neurogenic bladder, lower Vol, lower cQmax, and higher PVR at baseline significantly predicted satisfactory surgical outcome [odds ratio (OR) = 2.51, *p* = 0.029; OR = 0.99, *p* < 0.001; OR = 0.06, *p* = 0.002; and OR = 1, *p* < 0.001, respectively]. The multivariate analysis showed only Vol and PVR were the significant predictors for surgical outcome (OR = 0.99, *p* = 0.016; and OR = 1.00, *p* = 0.021, respectively) (Table 3).

**Figure 1:**
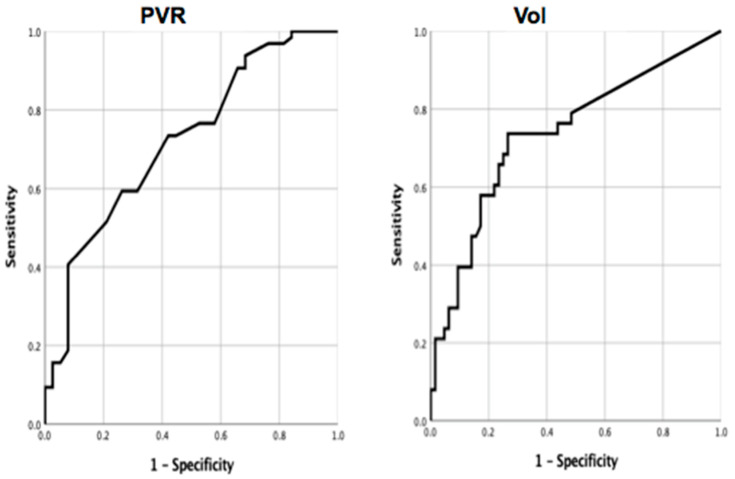
Receiver operating characteristic curves of baseline post-void residual urine (PVR) and voided volume (Vol.) for predicting satisfactory surgical outcome after transurethral incision of bladder neck. The area under the curve (AUC) values was 0.72 (95% confidence interval = 0.62 to 0.82) and 0.74 (95% confidence interval = 0.63 to 0.84), respectively. The optimal cutoff value of PVR was 335, with a specificity of 74.0% and a sensitivity of 59.0%, and of Vol. was 38 mL, with an acceptable specificity of 74.0% and sensitivity of 73.0%.

The receiver operating curve analysis of baseline Vol and PVR showed the AUC values were 0.74 (95% confidence interval= 0.63 to 0.84) and 0.72 (95% confidence interval = 0.62 to 0.82), respectively. (Figure 1) The optimal cutoff value of Vol was 38 mL, with an acceptable specificity of 74.0% and sensitivity of 73.0%; PVR was 335 mL, with a specificity of 74.0% and sensitivity of 59.0%. Age and underlying comorbidity such as coronary disease, congestive heart failure, diabetes mellitus, hypertension, and cerebrovascular accident showed no significant correlation to surgical outcome.

## 4. Discussion

Female voiding dysfunction is frequently encountered in urological practice due to neurogenic or non-neurogenic etiologies, including DU, BNO, or unknown causes [12]. Although CIC is the standard treatment option, patients usually wish to void spontaneously without the need for a catheter in order to have a better life quality. This study demonstrated TUI-BN alone or in combination with an additional procedure was safe, effective, with long-term durability in 62.7% of women. Patients with DU benefit most in resuming spontaneous voiding.

DU exists among the elderly resulting in chronic urinary retention, recurrent urinary tract infection, deteriorating renal function, and long-term catheterization, which affected the quality of life. Up to now, there is limited effective pharmacotherapy and a lack of consensus in treating DU patients [13]. TUI-BN has a promising high success rate of 74.6% to resume spontaneous voiding in DU patients and this therapeutic efficacy was similar to previous studies [9,14]. An adequate incision of the bladder neck smooth muscle until the serosa is crucial for a successful surgical outcome.

A wide-open bladder neck under cystoscopy; and the funnel shape of the bladder neck opening during the voiding phase effectively reduces the resistance of the bladder outlet. On the premise of adequate abdominal pressure, TUI-BN may facilitate spontaneous voiding in DU patients by abdominal straining resulting in increased VE and reduced PVR. Interestingly, this study revealed a dramatic improvement in Qmax by 413.3%, Vol by 346.7%, cQmax by 369.2%, BCI by 122.8%, and PVR by 68.7% in the successful group after treatment. TUI-BN alone or combining additional surgery provided a promising surgical outcome in patients with DU. Therefore, TUI-BN might be considered standard therapy for DU patients if medical treatment failed and patients desired spontaneous voiding without CIC.

Based on the analysis of urodynamic parameters, the study revealed a higher PVR and a lower voided volume at baseline are the predictive factors for a successful surgical outcome. Bladder neck plays a crucial role in the voiding phase. A high sympathetic tone of the bladder neck might lead to negative feedback that inhibits the detrusor contractility during the voiding phase [15]. In this study, the BCI improved by 122.8% once TUI-BN was performed. The previous study revealed an open bladder neck on voiding cystourethrography is the only predictive factor for successful urethral sphincter Botox injection in patients with voiding dysfunction regardless of whether neurogenic or non-neurogenic based [16,17]. Once the bladder neck was widely opened by TUI-BN but patients still had a suboptimal outcome, additional TUI-BN, TUI-ES, or urethral sphincter Botox injection could subsequently provide a satisfactory outcome with an additional success rate of 64.7% in those who failed the first TUI-BN.

A closed bladder neck and urethral opening were usually noted during the voiding phase in DU patients with or without neuropathy in VUDS. It is a challenge to distinguish between the association of inadequate detrusor contractility, BND, or tight urethral external sphincter, especially in DU patients during VUDS. The sympathetic nerve mediated by adrenergic alpha-receptors extends from the bladder neck and prostate to the external urethral sphincter in males [18]. Pudendal nerve stimulation evoked somatic responses in the external urethral sphincter and increased bladder neck pressure [19]. In this study, the success rate for TUI-BN alone particularly in DU patients was only 30.5%; however, the success rate can be raised to 76.5% with the combination of additional surgery. Therefore, in patients who failed the initial TUI-BN, a precise post-operative VUDS was mandatory and additional surgery was essential in order to resume spontaneous voiding.

In this study, 90.4% of patients with neurogenic bladder had the diagnosis of DU. The population with neurogenic bladder was younger in age, mostly due to post-pelvic surgery. Therefore, the patients usually had stronger abdominal pressure while voiding. The reduction in bladder neck resistance by TUI-BN and additional surgery enables spontaneous voiding in neurogenic bladder patients, with VE improved by up to 50%. Conversely, most of the patients with non-neurogenic bladder had a lack of abdominal straining ability, as they were frail and associated with multiple underline comorbidity

Patients with hypersensitive bladder had the lowest success rate of 22.2%. In a previous study, bladder pain caused bladder outlet dysfunction, and one-third of the patients were found to have a functional obstruction at the external sphincter. Chronic inflammation of the bladder might result in external sphincter hyperactivity while voiding [20]. Visceral pain syndromes might associate with central and peripheral sensitization and lowering of the nociceptive threshold, resulting in neuropathic upregulation, hypersensitivity, allodynia, and dysfunctional voiding [21]. Increased pudendal afferent activity through the guarding reflex of the external urethral sphincter, and thus inhibited the efficiency of bladder contractility [22]. The ongoing chronic inflammation sustained external sphincter spasticity in addition to inhibition of bladder contractility, leading to a less satisfactory surgical outcome despite undergoing TUI-BN or additional surgery.

The adverse events of TUI-BN and additional surgery were limited and confined to a certain population. Among the four patients who developed vesicovaginal fistula, three of the patients underwent TUI-BN twice and one of them underwent additional TUI-ES twice. Interestingly, all of them were associated with de novo incontinence and underwent suburethral sling surgery latterly and two of them underwent additional urethral sphincter PRP injection. Autologous PRP is rich in growth factors and cytokines, which regulate tissue reconstruction that augments wound healing, speed recovery from muscle and joint injuries, and enhance recovery after surgical repair [23]. PRP was injected at the urethral external sphincter in order to increase urethral resistance [24]. Three patients resumed spontaneous voiding and another one was under self-voiding and CISC. The confined patients underwent multiple repeated surgeries and complication-recovery surgery mainly due to unhealthy localized tissue and repeated surgeries causing tissue adhesion. In addition, 12 patients underwent suburethral sling surgery due to de novo incontinence after TUI-BN. All had recovered from stress urinary incontinence, eight patients were under self-voiding, three patients under CISC with self-voiding, and another patient under CISC.

The limitations of this study are its retrospective design and single-center experience. A prospective study should be designed to validate the effectiveness of TUI-BN in females with voiding dysfunction and a tight bladder neck.

## 5. Conclusions

TUI-BN alone or in combination with an additional procedure was safe, effective, and durable. Patients with DU have the best success rate in resuming spontaneous voiding after treatment. The lower voided volume and higher post-void residual volume showed a significant association for the likelihood of treatment success.

## Figures and Tables

**Table 1:** The baseline videourodynamic parameters and diagnosis of patients among successful and failed groups.

Parameters	Total (*n* = 102)	Fail (*n* = 38)	Success (*n* = 64)	*p*
Age (years)	61.17 ± 16.29	59.37 ± 16.17	62.23 ± 16.40	0.393
FSF (mL)	166.84 ± 80.50	149.42 ± 79.92	176.79 ± 79.74	0.104
FS (mL)	256.72 ± 107.36	230.17 ± 105.67	271.89 ± 106.18	0.063
CBC (mL)	423.95 ± 199.19	381.30 ± 188.09	449.00 ± 202.70	0.101
Pdet.Qmax (cmH_2_O)	17.81 ± 24.20	23.06 ± 22.55	14.81 ± 24.77	0.103
Qmax (mL/s)	3.71 ± 4.67	5.54 ± 4.74	2.63 ± 4.31	0.002
Vol (mL)	80.93 ± 113.65	139.66 ± 136.75	46.06 ± 80.05	<0.001
PVR (mL)	339.48 ± 202.92	242.63 ± 168.05	396.98 ± 201.03	<0.001
Compliance	64.69 ± 71.90	76.92 ± 99.33	57.69 ± 49.65	0.202
cQmax	0.20 ± 0.26	0.31 ± 0.29	0.13 ± 0.21	<0.001
BOOI	10.54 ± 24.38	12.28 ± 22.71	9.54 ± 25.41	0.593
BCI	35.99 ± 36.54	50.00 ± 36.11	27.98 ± 34.57	0.003
Pves (cmH_2_O)	64.31 ± 44.05	59.35 ± 46.00	67.07 ± 43.06	0.416
Pabd (cmH_2_O)	43.91 ± 52.74	33.03 ± 52.97	50.13 ± 52.00	0.121
VE	0.22 ± 0.28	0.39 ± 0.33	0.11 ± 0.17	<0.001
VUDS Diagnosis				0.022
Stable bladder	4 (3.92)	1 (2.63)	3 (4.69)	
BND	4 (3.92)	2 (5.26)	2 (3.13)	
DU	59 (57.84)	15 (39.47)	44 (68.75)	
DOLC	25 (24.50)	12 (31.58)	13 (20.31)	
HSB	10 (9.80)	8 (21.05)	2 (3.13)	

Data were expressed as mean ± standard deviation or number (percentage), BND: bladder neck dysfunction; DU: detrusor underactivity; DOLC: Detrusor overactivity and low contractility; HSB: Hypersensitive bladder; BOOI: bladder outlet obstruction index = Pdet.Qmax-2 × Qmax; BCI: bladder contractility index; CBC: cystometric bladder capacity; FS: full sensation; FSF: first sensation of filling; Pdet.Qmax: detrusor pressure at Qmax; Pves: vesicle pressure at Qmax; Pabd: abdominal pressure at Qmax; PVR: post-void residual; Qmax: maximum flow rate; Vol: voided volume; VE: voiding efficiency. *p* < 0.05 considered a significant difference.

**Table 2:** The changes of videourodynamic study parameters after surgery.

Parameters	Total (*n* = 102)	Fail (*n* = 38)	Success (*n* = 64)	*p*
FSF (mL)	−6.79 ± 109.05	17.68 ± 100.13	−25.91 ± 113.40	0.136
FS (mL)	−2.16 ± 141.05	25.92 ± 125.40	−24.09 ± 150.44	0.186
CBC (mL)	−89.30 ± 224.01	−35.88 ± 211.45	−121.89 ± 226.91	0.069
Pdet.Qmax (cmH_2_O)	−4.32 ± 24.98	−4.68 ± 21.78	−4.03 ± 27.57	0.924
Qmax (mL/s)	5.16 ± 8.53	0.56 ± 6.21	8.15 ± 8.54	<0.001
Vol (mL)	93.87 ± 169.26	−26.51 ± 95.06	159.71 ± 165.03	<0.001
PVR (mL)	−171.21 ± 239.43	−0.33 ± 181.04	−272.67 ± 211.16	<0.001
Compliance	2.68 ± 113.79	13.34 ± 163.58	−5.64 ± 50.08	0.537
cQmax	0.32 ± 0.55	0.08 ± 0.46	0.48 ± 0.55	<0.001
BOOI	−16.77 ± 33.15	−9.33 ± 32.21	−21.45 ± 33.24	0.138
BCI	17.71 ± 49.32	−8.81 ± 35.13	34.37 ± 49.96	<0.001
Pves (cmH_2_O)	−4.09 ± 50.79	−0.92 ± 54.77	−6.63 ± 48.18	0.685
Pabd (cmH_2_O)	−6.20 ± 54.79	4.82 ± 58.94	−13.73 ± 51.14	0.169
VE	0.33 ± 0.42	−0.08 ± 0.25	0.57 ± 0.29	<0.001

Data were expressed as mean ± standard deviation or number (percentage), BOOI: bladder outlet obstruction index = Pdet.Qmax − 2 × Qmax; BCI: bladder contractility index; CBC: cystometric bladder capacity; FS: full sensation; FSF: first sensation of filling; Pdet.Qmax: detrusor pressure at Qmax; Pves: vesicle pressure at Qmax; Pabd: abdominal pressure at Qmax; PVR: post-void residual; Qmax: maximum flow rate; Vol: voided volume; VE: voiding efficiency. *p* < 0.05 considered a significant difference.

**Table 3:** The univariate and multivariate analysis of predictor factors associated with success rate.

	Univariate		Multivariate	
Parameters	OR (95% CI)	*p*	OR (95% CI)	*p*
Age	1.01 (0.99 to 1.04)	0.390		
CAD	0.32 (0.07 to 1.44)	0.140		
CHF	1.19 (0.10 to 13.62)	0.887		
DM	1.52 (0.49 to 4.72)	0.466		
HTN	1.58 (0.68 to 3.68)	0.288		
CVA	0.88 (0.23 to 3.34)	0.850		
Neurogenic bladder	2.51 (1.10 to 5.73)	0.029	0.99 (0.36 to 2.73)	0.978
FSF	1.00 (1.00 to 1.01)	0.108		
FS	1.00 (1.00 to 1.01)	0.066		
CBC	1.00 (1.00 to 1.00)	0.106		
P_det.Qmax_	0.99 (0.97 to 1.00)	0.114		
Vol	0.99 (0.99 to 1.00)	<0.001	0.99 (0.99 to 1.00)	0.016
PVR	1.00 (1.00 to 1.01)	<0.001	1.00 (1.00 to 1.01)	0.021
Compliance	1.00 (0.99 to 1.00)	0.212		
cQmax	0.06 (0.01 to 0.35)	0.002	1.60 (0.14 to 18.93)	0.709
BOOI	1.00 (0.98 to 1.01)	0.591		
P_ves_	1.00 (0.99 to 1.01)	0.413		
P_abd_	1.01 (1.00 to 1.02)	0.125		
DOLC	0.49 (0.20 to 1.19)	0.116		
Outcome				

CAD: coronary artery disease; CHF: congestive heart failure; DM: Diabetes Mellitus; HTN: hypertension; CVA: cerebrovascular accident; FS: full sensation; FSF: first sensation of filling; CBC: cystometric bladder capacity; P_det_._Qmax_: detrusor pressure at Qmax; Vol: voided volume; PVR: post-void residual; Qmax: maximum flow rate; cQmax: corrected maximum flow rate; P_ves_: vesicle pressure at Qmax; P_abd_: abdominal pressure at Qmax; BOOI: bladder outlet obstruction index = P_det_._Qmax_ − 2 × Qmax; VE: voiding efficiency; DOLC: detrusor overactivity and low contractility. *p* < 0.05 considered a significant difference.

## Data Availability

Data request are available by contacting with the corresponding author.
